# Cost Benefit Analysis of Two Policy Options for Cannabis: Status Quo and Legalisation

**DOI:** 10.1371/journal.pone.0095569

**Published:** 2014-04-22

**Authors:** Marian Shanahan, Alison Ritter

**Affiliations:** National Drug and Alcohol Research Centre, UNSW Australia, Sydney, Australia; University of Milan, Italy

## Abstract

**Aims:**

To date there has been limited analysis of the economic costs and benefits associated with cannabis legalisation. This study redresses this gap. A cost benefit analysis of two cannabis policy options the status quo (where cannabis use is illegal) and a legalised–regulated option was conducted.

**Method:**

A cost benefit analysis was used to value the costs and benefits of the two policies in monetary terms. Costs and benefits of each policy option were classified into five categories (direct intervention costs, costs or cost savings to other agencies, benefits or lost benefits to the individual or the family, other impacts on third parties, and adverse or spill over events). The results are expressed as a net social benefit (NSB).

**Findings:**

The mean NSB per annum from Monte Carlo simulations (with the 5 and 95 percentiles) for the status quo was $294.6 million AUD ($201.1 to $392.7 million) not substantially different from the $234.2 million AUD ($136.4 to $331.1 million) for the legalised–regulated model which excludes government revenue as a benefit. When government revenue is included, the NSB for legalised–regulated is higher than for status quo. Sensitivity analyses demonstrate the significant impact of educational attainment and wellbeing as drivers for the NSB result.

**Conclusion:**

Examining the percentiles around the two policy options, there appears to be no difference between the NSB for these two policy options. Economic analyses are essential for good public policy, providing information about the extent to which one policy is substantially economically favourable over another. In cannabis policy, for these two options this does not appear to be the case.

## Introduction

An estimated 128.9 million to 190.7 million people worldwide used cannabis in 2010 [1]. This consumption occurs while the supply and often the possession of cannabis remain illegal although some jurisdictions have introduced alternate regulatory strategies such as *de facto* regulation coffee shops in the Netherlands [2]; Commissions for the Dissuasion of Drug Addiction in Portugal [3]; civil penalties for possession and use of cannabis in several states in Australia [4] and the use of cannabis for medical reasons in the United States [5]. These regulatory strategies, often referred to as decriminalisation, do not legalise either possession or supply. To date no country has fully legalised the possession and supply of cannabis, although we note the recent move to legalising cannabis in the states of Colorado and Washington, USA and in Uruguay, a bill to legalise cannabis is before the Senate. This places an analysis of the costs and benefits of cannabis legalisation at the forefront of drug policy.

Advocacy for the legalisation of cannabis has been well-documented [6], [7], [8], [9] as have the arguments against [10], [11]. Advocates for legalisation argue that maintaining the criminal status of cannabis encourages criminal activity, necessitates contact with illicit drug sellers, leads to individuals acquiring criminal records for possession of small amounts of cannabis, results in taxation losses, and increases the costs of enforcement. Advocates of total prohibition argue that prohibition leads to lower consumption of cannabis, better health status, and improved productivity.

The need for a systematic assessment of drug policy options and their consequences are outlined elsewhere [5], [12], [13], [14], [15]. While the importance of the role of economic evaluations in such a systematic assessment has been recognised, to date there has been no quantification of the significant costs and benefits of one legal option over the status quo. Past research has examined selected outcomes of a policy change; for example, the cost impacts on the criminal justice system [9], [16], [17], [18]; gains in taxation income [9], [16]; impacts on use [16], [19], [20]; on educational attainment [21], [22], [23]; and on driving [24], [25]. However, to our knowledge no previous research has combined and valued the wide range of costs and benefits of cannabis policies, nor included individuals’ costs and benefits.

This study seeks to begin to address this gap in knowledge. It reports on the results of a static cost benefit analysis (CBA) of two policies for cannabis over a one year time frame. CBA uses a common monetary metric to value both the costs and benefits making it particularly useful when there are multiple and/or conflicting outcomes of a policy [26], [27].

A CBA is conducted in a normative framework which explicitly assesses the costs and benefits of social policies [26] across multiple domains. CBA has its foundations in welfare economics, the branch of economics that addresses normative questions (what should be) as compared to positive economics (making predictions without value judgements). Normative, in the context of CBA, assumes that: i) social welfare is made up from the welfare (or utilities) of each individual within society and ii) individuals are the best judges of their own welfare (consumer sovereignty) [28].

The central concept that lies behind CBA is Pareto optimality which is defined as ‘no person should be worse off under an alternative program compared to prior to its introduction’. However, the consequence of strict Pareto optimality is that it would be virtually impossible to introduce a program or policy which was deemed beneficial to society. For example, the movement to the alternative program, say legalisation of cannabis, would not be considered a Pareto optimal solution if the program resulted in a large number of individuals each gaining considerable benefits [29] (i.e. the freedom to use cannabis legally) while losses ensued to a small number of individuals (i.e. additional adolescents not achieving their maximum level of education because they were enticed to use cannabis at a young age). In response to this issue, in the late 1930s, economists noted that the important issue was aggregate real income (benefits) – that is, it is not about each individual but rather that some people could be made better off without making society worse off [30]. This criterion, referred to as the Kaldor-Hicks criterion does not mean redistribution from winners to losers actually takes place but merely that it could. If the criterion is passed then the project is determined to be allocatively efficient.

For some individuals within society, greater harm may result from a policy change, whereas for others there will be reduced harm. A CBA provides an analysis ‘net’ of the gains and losses to sectors or individuals in society. This would suggest that an increase in harm to some individuals due to increased use of cannabis following a policy change is neither a necessary nor sufficient condition for that policy to be socially undesirable if the overall sum of the positive benefits outweighs the sum of the costs and loss of benefits. This is an important point, ignored by much of the clinical research literature, which concentrates on estimating the magnitude of individual harms, and often appears to assume that the existence of such harms necessarily generates a case for prohibition [31]. The collation and valuation of the costs and benefits in a CBA allows comparison and ranking of the results of the various policy options [32]. To avoid the problems that arise when arbitrary decisions are made as to whether a certain item is a cost, a saving, or a benefit, the results are reported as a net social benefit (NSB) [28], [33] thus avoiding the debate as to whether something is a disbenefit or a cost. The NSB is the sum of all the valued benefits (b) minus the sum of all the valued costs (c). Once the NSB is calculated, all those policy options where the NSB is greater than zero are ranked according to their NSB from lowest to highest, with the preferred option being that with the highest NSB.

Key challenges in conducting a CBA are in the measurement and quantification of the costs and benefits, both the tangible and intangible. Many so-called CBA simply quantify the implementation costs and savings. One review of the CBA literature reported that 60% of the studies claiming to be a CBA were actually costing studies which ignored potential benefits (lost or gained) [28]. The results of the CBA do not provide a true societal valuation of a policy if significant harms and benefits of an intervention are excluded.

The two policies assessed here are the current policy (status quo) in New South Wales (NSW), Australia and a modelled, highly regulated–legalised policy. As cannabis policies vary across Australia, it was necessary to choose one jurisdiction and NSW is the most populous. In NSW while cannabis is illegal there are diversion programs such as cannabis cautioning for the possession of a small amount of cannabis for adults and warnings for juveniles [34], [35]; neither of which result in a criminal record. This represented the status quo model.

As there is no single agreed upon model for legalising cannabis and none of the proposed models in the literature had been implemented when this research was undertaken [36] a legalised–regulated policy option was constructed based on the public health approach articulated by Nadelmann [37] and further expanded by others [14], [38], [39]. The key characteristics of the legalised–regulated policy includes licensing consumers, cannabis only retail shops, disallowing promotion and advertising [38], [40], monopoly distribution and retail [38], age restrictions; restrictions on location of consumption [41] and pre-negotiated purchase contracts with growers [42]. Further details of the status quo and legalised–regulated model are summarised in Table 1.

**Table 1:** Characteristics of the two policies.

Illegal with diversion programs	Legalised and regulated
Cannabis is illegal	Positive consumer licence
Formal cannabis caution available:	Age 21 years
*Adults*	Legal to grow 10 plants for own consumption
2 cautions permitted for possession of less than 15 grams of cannabis	Operating vehicles under the influence illegal
Court diversion into treatment program available	Limits on location of consumption
*Juveniles*	Monopoly distributor (not-for-profit)
Informal warnings	Grower’s permit required & re-negotiated forward contracts Record keeping (type of seed, OHScompliance workers training etc)
Formal cautions	Potency tests (recorded on packaging)
Juvenile conferencing	Cannabis & implements only retail shops
Fines and/or imprisonment for supply and cultivation of a trafficable amount	Limited density of shops
	No advertising, plain paper packaging, health warnings
	Sale price set by regulatory board Regulatory board responsible for enforcing regulations

## Methods

Ethical approval for this work was provided by the University of New South Wales Human Research Ethics Committee.

The costs and benefits of each policy option were classified into the following five categories [43]: i) direct intervention costs e.g. enforcing the laws or regulations; ii) costs or cost savings to other agencies, individuals or families e.g. treatment for dependence; iii) benefits to the individual or the family from the policy; iv) externalities which are the unintended effect on a third party such as change in productivity or injuries to third parties; and v) adverse or spill over events. Table 2 lists the components of each category included in this study. All costs and benefits are expressed in 2007 Australian dollars (AUD).

**Table 2:** Costs and benefits included the model.

Direct intervention costs	Costs or cost savings forother agencies, individuals,and families	Benefits lost or gainedfor the individualor family	Externalities
Criminal justice system	Health care costs	Impact on number of persons with criminal record & potential stigma from criminal record	Accidents/injuries to third parties as aconsequence of increased cannabis use
• Police	• Cannabis treatment	Value of the enjoyment from cannabis use	Increased use of tobacco
• Courts and court diversion program	• Other health consequences	Impact on educational attainment and subsequent earnings	Attitudinal changes: cannabis usebecomes more acceptable –use increases
• Prosecution/Legal Aid	Prevention programs		
• Corrective services	Personal		
Grower	• Fines		
• Growers permit	• Legal defence costs		
• Legal costs to negotiate contract	• Parents’ lost productivitywhen attending court		
• Cost of complying with NSW workplacelaws and agricultural regulations			
• Testing for potency			
Distributor/retailer			
• Infrastructure costs			
• Staffing/training			
• Transportation			
Consumer			
• Licence/course			
Enforce regulations			
• Police			
• Regulatory body (licensing/standardsetc.)			
• Contract negotiation			
• Black market			
• Drug driving testing programs			

Although there continues to be some debate in the literature as to whether or not the dis/benefits to those who have committed a criminal offence should be included in a CBA [44], [45] it does not seem compelling to *not* include the dis/benefits accrued by the individuals who are currently being found guilty of cannabis offences (and in some circumstances incarcerated) in a study comparing policy options for cannabis. To be clear it is only the benefits related to cannabis offences/offenders which are included.

A range of methods were used to quantify and value the various inputs and benefits using both primary and secondary data. (Further details on estimating costs and benefits are found in Shanahan [46]). As the intent of this study was to provide experimental analyses of the costs and benefits (rather than calculations that government would use to judge costs/savings) the comparison between the two policy options - the status quo and the hypothetical legalised-regulated – are presented as total costs and benefits. An alternative presentation would have been to present the marginal costs and benefits (that is only the change in costs and benefits for the hypothetical policy option relative to the status quo). Given one of the objectives of this work was to compare the two options directly we chose to present the total (not marginal) costs and benefits for both.

The criminal justice system costs in the status quo option included the costs of policing (including warnings and cautions), courts, prosecution, legal aid, and corrective services for those who were detected by police with cannabis. These costs were estimated from a survey of police activities conducted for this study; one year of police data on cannabis offenses by type of offence, unit record court data for NSW (frequency, outcome and sentence by offence type); data on court activity costs; the budget for Department of Public Prosecution and Legal Aid (pro rata by offences) and court diversion program data [47], [48], [49], [50]. Wages lost during imprisonment for cannabis offenses were estimated by multiplying the number incarcerated by the average duration of the sentence for each type of offence and court. This in turn was multiplied by the 2007 minimum wage [51]. These estimates included only potential lost wages and did not include any valuation of the loss of personal time while incarcerated. Also included was the value of fines paid for cannabis offences by cannabis offence and by court type obtained for one year.

Stigma from a criminal record due to cannabis offences was valued and included in the status quo. The willingness to pay to pay to avoid stigma valuation was obtained from a community sample of 875 persons and was then applied to the number who were convicted of a criminal offence in one year who did not have a pre-existing criminal record [52].

Under the legalised–regulated model the policing costs associated with cannabis use/possess arrests and police actions were excluded as these were no longer applicable. The value of stigma from a criminal record was also not included in the legalised–regulated model as it was also not applicable. Under the legalised–regulated model, the criminal justice system costs included the enforcement of regulations, offenses of underage use and supply, and ongoing use of some existing services. For example, the NSW court diversion program for cannabis offenders is not likely to disappear under legalisation. Currently, of those who attend the three month court diversion treatment program for those with a demonstrated drug problem (including alcohol) and report cannabis as their drug of concern, only 35% of those had a cannabis offence as their principal offence [50]. In the absence of other information, 65% of the status quo expenditure for cannabis diversion was included under the legalised–regulated costs. The impact of this decision was tested through sensitivity analyses.

Health care costs for the status quo were sourced from previous research in NSW [53] and included annual total costs for treatment for cannabis use disorder (CUD), schizophrenia and psychosis, low birth weight newborns [54], [55], [56] and motor vehicle accidents [57], [58], [59]. While the debate on the precise relationship between cannabis use and schizophrenia and/or psychotic disorders is ongoing, there does appear appears to be some association. The number of persons with cannabis related schizophrenia was estimated using a population attributable fraction (PAF) calculated using odds ratios (ORs) [60], and the number of cannabis users who were treated for and/or diagnosed with schizophrenia/psychotic disorders in last 12 months [61] with adjustments for frequency of use. The resulting numbers were then multiplied by the costs of treatment and diagnosis [62], [63]. (See [53] for more details). These healthcare costs were applied to the status quo model.

For the legalised–regulated model, healthcare costs were estimated based on the number of cannabis users in the legalised–regulated model. The prevalence and frequency of cannabis consumption for the status quo model was obtained from the 2007 National Drug Strategy Household Survey (NDSHS) data [61] with data from international research [64], [65] used to generate ranges for the sensitivity analyses. Additional data on amount consumed per use day and grams per joint were also sourced from the NDSHS. Estimates of prevalence and quantity consumed under the legalised–regulated model were then estimated from the responses to the question in the NDSHS (2007) “if cannabis/cannabis were legal to use, would you (not use, decrease, try it, increase, or not change use)?” Starting from the existing use patterns, those who stated they would increase their use were shifted up one use category (i.e. monthly users were changed to at least weekly, at least weekly were changed to daily use); those who were not current users but stated they would ‘try cannabis’ were distributed according to existing overall use patterns. Under these assumptions the prevalence of cannabis use increased by 44% to a population rate of 12.4% and consumption increased by 55%.

These new prevalence figures, by type of use, were applied to estimate many of the costs and benefits under the legalised–regulated model. For example, to estimate the potential additional costs due to schizophrenia or psychosis the additional number of heavy and light users was multiplied by the number of cannabis users (heavy and light) that must be prevented (NNP) in order to prevent one case of schizophrenia and one case of psychosis [66] giving the potential additional number of new cases of psychosis (n = 55) and schizophrenia (n = 24). These numbers were then multiplied by the relevant average costs [53].

In order to estimate the additional numbers with cannabis use disorder the current population rates (with standard errors) of cannabis use disorder for Australia [67] was multiplied by the NSW population. The rate was then estimated among those in NSW who used cannabis on more than five occasions in the past 12 months. This rate was then applied to the total estimated number of cannabis users who would consume cannabis on more than five occasions in one year under legalisation-regulation. This results in a 37% potential increase in the numbers seeking treatment for CUD. This relies on the assumptions that the association between cannabis use disorder and treatment, and types of treatment remain constant, and that any increased demand for treatment would be met.

Although there have been equivocal findings on the effect of cannabis on driving [5], a recent review of the literature concluded that cannabis has a collision-enhancing effect [68]. Estimates from Ngui (201) were used for the number of accidents attributable to cannabis use. For the regulated-legalised model, the assumption was made that the number of accidents increases with not only the increase in prevalence of use, but also with the frequency of use. Here the number of total days of consumption in each of the two methods was used. The probability of a fatality [57], [69], given the number of days of cannabis consumption was applied to the estimate of projected changes in days of use. This analysis was then repeated for minor, major and permanent injuries. In summary, the probability of a fatality given the number of days of cannabis consumption (current) was applied to the estimate of projected changes in days of use to derive the number of fatalities, minor, major and permanent injuries. With these assumptions, the increases in fatalities range from one to four, and serious injuries increase by ten to fifty in a year. For both the status quo and legalised–regulated model, the statistical value of a life year lost was sourced from the existing literature [70]. The costs of the injuries were sourced from Ngui (2010).

Cannabis acutely impairs cognitive performance [12] but whether there is a lasting cognitive impairment of the still-developing brain when cannabis use begins in adolescent years continues to be debated. The potential impacts of cannabis consumption on educational attainment in both the status quo and the legalised–regulated models were estimated by combining the prevalence and consumption data (as described earlier) with estimates of the negative impact of cannabis consumption on educational attainment [21], [22], [23]. A subsequent potential loss of income was obtained by multiplying the estimated years lost by 10% (range 8% to 12%) of average earnings that is gained for every additional year of schooling [22], [71].

Typically economic evaluations conducted in the illicit drug and alcohol fields have not recognised any positive utility (wellbeing) from the purchase and consumption of tobacco, alcohol or illicit drugs [72], [73] but it is often argued that any positive utility from consumption must be included [26], [74]. Cannabis users when asked why they consume cannabis provide a number of reasons such as it helps them to relax, to get intoxicated, to socialise, it enhances some activities, lessens boredom, and aids in sleep [75]. Thus to the consumer, the consumption of this drug provides some change in wellbeing. Without some measure of wellbeing, any valuation of the legalisation of cannabis would be undervalued.

The problem is that there is no monetary metric to quantify the change in wellbeing. We also do not know the shape of the demand curve for cannabis [16]. In the absence of this knowledge [16], we used as a proxy the change in the value of the quantity of cannabis consumed (holding the price constant). The assumption here is that the demand curve shifts outwards with legalisation. The change in the wellbeing gained by consumers’ from cannabis consumption was estimated through the use of the current prevalence and consumption measures and the new estimates for legalisation as described earlier. For the purpose of quantifying wellbeing, consumption by those with a cannabis–use disorder or under the age of 21 was excluded. The proxy for wellbeing was then estimated by multiplying consumption by the median street price of cannabis in NSW [76].

The costs for the legalised–regulated policy option were estimated given the features of the model as described in Table 1. The regulatory framework included a licensing program as one way of controlling cannabis tourism, and to ensure those purchasing cannabis were aware of potential harms and where to seek help if required. The cost per consumer to obtain a licence ($45 per year) was based on the NSW Driver’s Licence program [77]. As a comparison the cost of the current state portion of a California Medical Marijuana Identification Card is currently $66USD plus individual counties may add administration fees and the consumer must pay for a visit to the medical doctor [78].

The cost of non-compliance to regulations (i.e. selling or providing to those less than 21 years of age, disregarding where cannabis can be consumed) was estimated based on the value of the penalties and their frequencies obtained from alcohol and tobacco data [48], [79]. The costs to the grower of complying with the regulation included testing for potency, employee training, and licences. The annual budget for the state of Tasmania’s Poppy Advisory and Control Board, the agency responsible for monitoring the legal production of poppies for morphine in Australia, was used as a starting point for the cost of the regulatory agency. The cost estimate was increased by 50% to account for a larger geographical area of the state of NSW and the fact that, unlike poppies, cannabis is cultivated year round in greenhouses. The cost of enforcing regulations and providing consumer information, quit programs and support were extrapolated from recommended per capita expenditures for tobacco control in Australia [80]. These estimates were adjusted for the expected prevalence of cannabis use in the population (12.4%) compared to that of tobacco (19.4%) [81].

Distribution and retail of cannabis in this model occurred through a government monopoly although another form of non-profit monopoly would achieve the same purpose. A monopoly structure was used as one way of countering claims of anti-competition by potential profit making businesses given advertising was not permitted and plain paper packaging would be required. The costs of operating the retail enterprise were based on the presumption of 330 cannabis shops across the state (two in every council area) selling only cannabis or cannabis implements; and operating for 12 hours per day, and staffed with two persons. Wages were sourced from the NSW Public Employees Award [82]. Payment to the contracted growers was based on a two-to three–and–half–fold difference in farm gate to retail prices [83]. The grower would receive approximately $5.50 to $8.80 per gram (equates to $35 to $72 million per year per one acre greenhouse).

Cannabis price was held constant in the model. With this strong and potentially unrealistic assumption (discussed further below), all change in demand for cannabis was attributed to the change in legal status (a shift in the demand curve).

How best to manage the income generated from revenue associated with the sale of cannabis under the legalised–regulated model required some thought. In a CBA, benefits normally only accrue to consumers and producers, or to third parties through externalities. If a policy change affects government revenues indirectly through changes in tax revenues, welfare payments, or subsidies they are normally ignored as they are considered transfer payments which do not affect overall net economic benefit [33]. When moving from a situation where the revenue from cannabis sales will accrue to governments rather than being captured by the illicit drug market it could be argued that some of these revenues are a gain to society and they should be included. The results are presented both without and with potential government revenues included in the legalised–regulated model.

The assumption of a constant price and the likelihood of a continuing black market for cannabis as well as home growing generated considerable uncertainty around the estimate of government revenues. Based on estimates of the size of the black market for tobacco in Australia [84] and current reported home cultivation of cannabis [61] it was assumed that 15% (range 10% to 20%) of potential revenue to government would not be collected. Others have projected larger losses as a result of the black market particularly if attempts are made to retain prices at current levels [9], [16] which if true would result in less government revenues.

Once the values were obtained for each component, the costs and benefits were summed to generate the NSB for each policy.

NSB Status quo = [value of stigma from criminal record _SQ_ *+*value of lost educational attainment*_SQ_+*value of wellbeing from consumption of cannabis*_SQ_*+lost wages while incarcerated*_SQ_ +*value of lost lives from accidents*_SQ_* - (costs to criminal justice system*_SQ_* i.e. *police, courts, penalties/prisons, MERIT testing for drug driving*) - (costs of health services*_SQ_* i.*e. treatment for dependence, mental health, low birth weight newborns, MVA accidents)*].

NSB Legalisation and regulation = [value of lost lives from accidents_L–R_ *+*value of lost educational attainment_L–R_ *+*value of wellbeing from consumption of cannabis_L-R_ -(costs to criminal justice system _L-R_ i.e. *MERIT_ L–R_, prosecution and fines for selling to underage_L–R_, testing for drug driving_L–R_*) - (costs to regulatory system_L-R_ i.e. *regulatory agency, enforcing regulations, consumer information, and quit campaigns*) - costs to the health care system as function of use_L-R_ - costs of retail operation_L–R_].

An additional design feature, which some may regard as a limitation, is that the comparison involves an *ex post* alternative (the current status quo) and an *ex ante* alternative (the hypothetical). It was not possible to conduct a fully *ex post* analysis, as cannabis legalisation had not been introduced. Conducting a full *ex ante* comparison, while possible would have entailed establishing a hypothetical status quo. This would have been somewhat counterintuitive to the audience of policy makers, who consider what currently exists and the possible alternatives.

In addition to the primary estimates a range was constructed for every variable using credible assumptions. Where none were available, a range of +/−20% was used. All costs were in 2007 Australian dollars. Monte Carlo simulation with a normal distribution and 1000 repetitions was conducted to generate the 5^th^ and 95^th^ percentiles around the mean.

## Results

The total cost pertaining to the current NSW policy was estimated at $80.1 million per annum (p.a.) (range $54.2 to $113.4) and $90.7 million p.a. ($53.8 to $128.8) for the legalised–regulated alternative (see Table 3). The specific costs for each component are detailed in Table five. As can be seen, under the status quo the largest single expenditure was on criminal penalties, followed by policing costs. Under legalisation–regulation the largest expenditure was personal costs of licensing ($31.1 million p.a.; range $23.3 to $38.9), followed by consumer information and QUIT campaigns ($12.5million p.a.; range $5.9 to $18.8). Treatment and other health care costs also increased under the legalised–regulated model from $6.8 million p.a. (range $6.2 to $7.5) to $10.8 m (range $7.4 to $12.5).

**Table 3:** Summary of annual total costs, total benefits and net revenues for both models and the Net Social benefit (AUD 2007).

	Status quo			Legalised – regulated		
	Main estimate(millions)	Low rangemillions	High rangemillions	Main estimate(millions)	Low range(millions)	High range(millions)
Total costs	$80.1	$54.2	$113.4	$90.72	$53.8	$128.8
Total benefits	$362.7	$282.1	$513.0	$318.8	$222.4	$394.2
Net government revenue				$659.4	$829.5	$196.5

The total benefits are estimated at $362.7 million p.a. (range $282.1 to $513.0 million) for the status quo and $318.8 million p.a. (range $222.4 to $394.2 million) for the legalised–regulated alternative. Both wellbeing from cannabis use and the value for decreased educational attainment were larger in the legalised–regulated model due to increased consumption of cannabis (detail is found in Table 4).

**Table 4:** Net social benefit in millions AUD from the Monte Carlo simulation (mean, 5th and 95^th^ percentile).

	Status quo			Legalised – regulated		
	Mean	5^th^	95^th^	Mean	5t^h^	95^th^
Costs and revenues from retail excluded	$294.6	$201.2	$392.7	$234.2	$136.4	$331.1
Costs and revenues from retail included				$727.5	$372.3	$1,113.2

**Figure 1:**
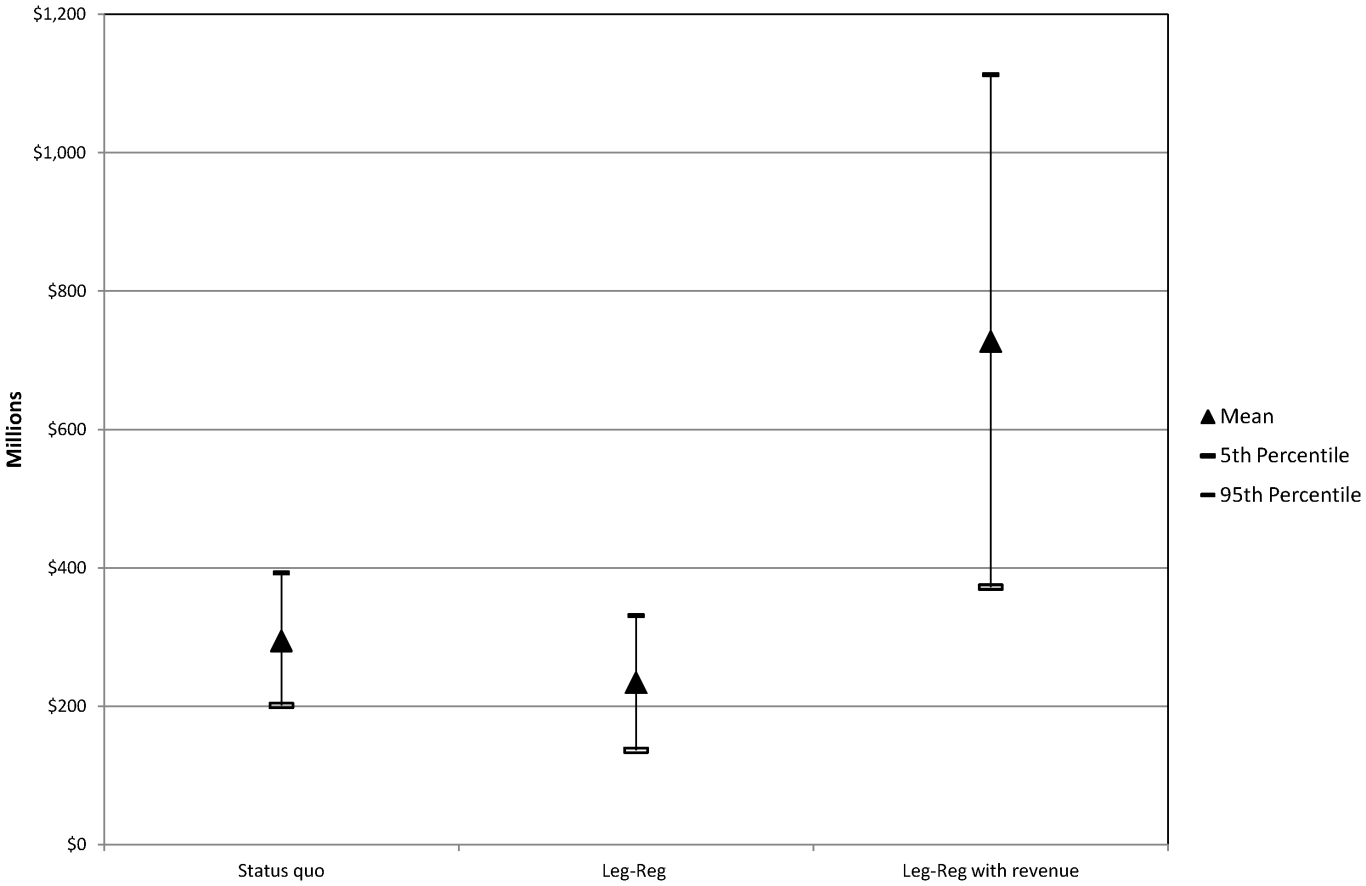
NSB main estimates from Monte Carlo simulation.

The mean NSB for the status quo was $294.6 million p.a. ($201.2 to $392.7 million) and $234.2 million p.a. ($136.4 to $372.3 million) for the legalised–regulated model (Table 4). There appears to be no substantive difference between the NSB for these two policy options. The results are presented in Figure 1. The addition of government revenues results in a larger NSB for the legalised–regulated model. However, the level of uncertainty around the results also increases. An important caveat is that this assumes all revenues which go to government are new revenues, i.e. no portion of the revenue from the illicit cannabis market returns to government as revenue under the status quo.

**Figure 2:**
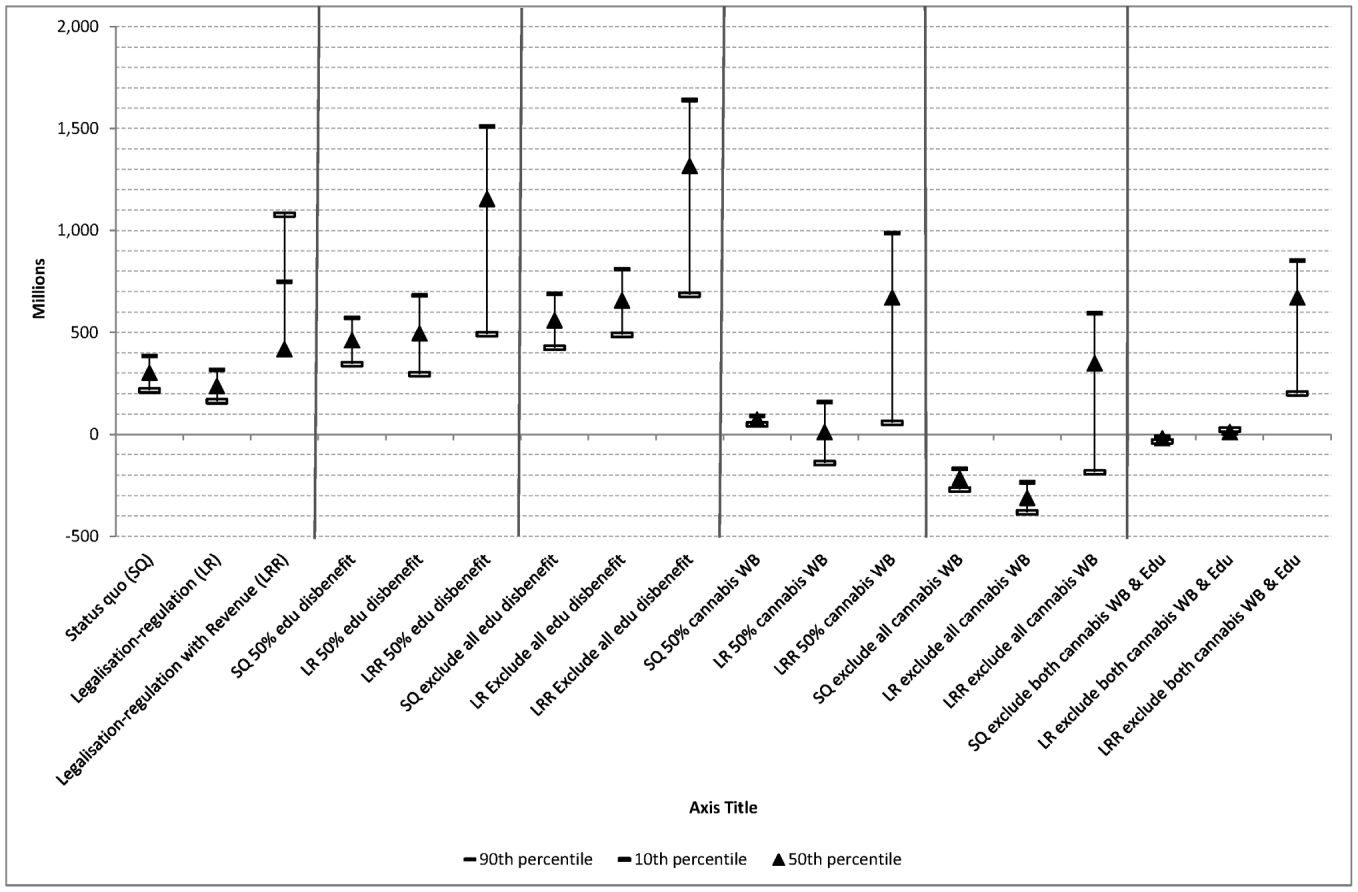
Examining impact on the NSB of various assumptions regarding benefits (millions $).

Figure 1 demonstrates the uncertainty around the main estimate using ranges from Tables 4. In addition, because of the potential influence on the results and uncertainty in measurement, the impact of varying two key benefits (wellbeing and educational attainment) was further explored in the sensitivity analyses. In Figure 2, the impact on the NSB of first decreasing educational attainment and gain in wellbeing by 50% and then removing them completely are demonstrated. All other benefits and costs were held constant in the simulation. Not surprisingly, the NSB increased when the negative impact of cannabis on educational attainment was halved, or removed completely. When the value for the wellbeing gained from cannabis was decreased to 50% or removed completely, the NSB for both the status quo and legalised–regulated alternative became negative as they did when the values for both educational attainment and wellbeing were excluded. That is, when the value attributed to personal wellbeing from cannabis is decreased the results indicate that neither policy is an efficient use of society’s resources.

Varying the costs within the ranges presented in Table 5 resulted in minimal changes to the NSB (ranging from a 6.4% increase in the NSB if the low range for penalties was used, and a 6% increase if the highest range for fines was used). All other cost categories had less than a 5% impact on the NSB.

**Table 5:** Summary of annual costs and benefits* (AUD 2007).

	Status quo			Legalised–regulated		
	Main	Low	High	Main	Low	High
Expenditures	*Millions $*			*Millions $*		
Police (including drug driving testing) costs	22.1	11.76	31.98	11.5	2.3	18.4
Court	4.8	3.6	6.16			
Prosecution and Legal Aid	3.2	0.7	5.8			
Penalties/Corrections	27.0	20.2	33.7			
Magistrates Early Referral into Treatment (MERIT	3.5	2.64	4.4	2.5	1.9	3.1
Regulatory agency				1.0	0.7	1.4
Enforcing regulations				4.0	1.4	6.5
Consumer information, quit campaigns and support				12.5	5.9	18.8
Treatment (CUD)	6.8	6.2	7.5	10.8	7.4	12.5
Schizophrenia/psychosis	6.2	4.7	8.1	7.1	5.3	9.2
Low birth weight newborns	1.6	0.5	5.9	2.9	0.5	7.3
Motor vehicle accidents	2.3	2.2	6.9	3.7	2.6	8.3
**Total government costs**	**77.6**	**52.4**	**110.3**	**55.9**	**27.9**	**85.4**
Fines (current)	1.3	0.9	1.6			
Parents lost work time	0.1	0.04	0.07			
Defence attorney	1.2	0.9	1.6			
Personal Licence costs				31.1	23.3	38.9
Fines under regulatory structure				3.2	2.2	4.0
**Total personal costs**	**2.5**	**1.9**	**3.2**	**34.3**	**25.5**	**42.9**
**Growers - compliance costs**				**0.5**	**0.4**	**0.5**
**Total costs**	**$80.1**	**$54.2**	**$113.4**	**$90.72**	**$53.8**	**$128.8**
**Dis (benefits)**						
Lost wages - incarcerated	-$8.7	-$6.5	-$14.5			
Stigma	-$7.4	-$1.3	-$15.1			
VOSLY (accidents)	-$3.0	-$2.3	-$3.8	−4.75	−3.38	−5.94
Wellbeing value	579.1	434.3	$723.9	645.5	484.2	786.8
Education attainment	−197.3	-$157.8	-$236.7	−323.0	−258.4	−386.7
**Total Benefits**	**$362.7**	**$282.1**	**$513.0**	**$318**	**$222**	**$394**
**Retail operation**						
Payments to growers				−617.5	−790.2	−385.9
Operating shops				−82.86	−450.1	−47.74
Total revenues				1,360	1,437	1,263
**Potential net revenue**				**$659.5**	**$196.5**	**$829.5**

*due to rounding not all totals may not sum exactly.

## Discussion

In undertaking a cost benefit analysis of the impact of two policies for cannabis this study has gone beyond the common evaluative approaches in cannabis research. Here, the costs to the criminal justice system and the potential costs of establishing a regulatory framework, of providing health care in response to cannabis induced harms, personal costs and economic benefits such as stigma, wellbeing and impacts on educational attainment are captured.

The two policy options analysed represent the current policy in New South Wales, one Australian jurisdiction, and a modelled public health approach (legalised and regulated) that was constructed with the objective of minimising harms both from the policy itself and from cannabis consumption. In presenting the total costs and benefits for the two policy options, the base to which each policy option was compared to was no cannabis consumption. Such an assumption, which also meant there was no change in consumption of an alternative product, is obviously not a realistic one but it did provide a common comparison for the two policies of interest. One limitation of the study design was the use of an *ex ante* policy with an *ex post* comparison. This was done because our purpose was to conduct an experiment with useful intuition for policy makers, but not to demonstrate how much governments might save (or loose) under the options. If governments were to consider cannabis legalisation, the next step would be to conduct a marginal analysis.

The CBA produced a positive net social benefit (NSB) for each of the policy options. There appears to be no substantial difference between the NSB for the status quo in NSW and the NSB for a legalised–regulated alternative (excluding potential revenue to government). This suggests that the policy alternatives are similar in their efficient use of society’s resources. The simple addition of the revenues to government, although not normally included in CBA, increased the NSB making the legalised–regulated option appear attractive. The confidence intervals increased substantially demonstrating the additional uncertainty, and given uncertainties around the price under legalisation [16], [36], the confidence intervals could be considerably wider. Both policies result in significant opportunity costs. For the status-quo costs are incurred by the criminal justice system (police, corrections and to a lesser degree in providing health care) while in the legalised–regulated model costs are more widely dispersed across policing (drug driving testing), prevention and education services, health care services and to individuals for the purchase–licence program.

The task of conducting a CBA where one policy is highly speculative and the good itself is illegal required many assumptions. In the complex area of illicit drugs policy the list of possible inputs, harms and benefits is long [12], [13], [43] and despite the extensive cataloguing of costs and benefits there were exclusions. Key in these were the potential impact on family members; the costs of combating gang related activities; the potential political consequences to those advocating for or against a change in policy; the potential effects on alcohol consumption if cannabis were to be legalised; and the impact on international relationships should cannabis be legalised. All of these categories were excluded due to the lack of available data. In NSW the focus of most gang related activity is not cannabis (personal communication, NSW Drug Squad). Additionally, based on the costs of combating gang-related activities for the illicit sales of tobacco [84], even under legalisation gang related activity would likely continue to some degree. With respect to the impact on alcohol consumption, currently the literature is in disagreement [85], [86] as to whether alcohol and cannabis are substitutes or complements and, even if this was known with certainty, it is not known whether the current relationship would persist if cannabis was legalised. Similarly, the issue of potential gateway effect of cannabis into other ‘harder’ drugs was not included. Room et al. (2008) pointed out that opportunity does play a role in other drug use, but that social environment, peer groups and delinquent behaviours do not explain all the relationships between cannabis and other drug use [5]. Limitations for any CBA are the exclusion of some variables, and further research should attempt to include these costs and benefits.

The quantification of costs and benefits required assumptions around the likely uptake of cannabis if it were legalised. Although quantity of cannabis consumed increased by 52% with legalisation the estimated 44% increase in prevalence is similar to that projected by others when prices are held constant [87], [88]. The quantity increased more than the prevalence as some who were already consuming cannabis under the current laws consume more under legalisation. Similarly, the estimates of wellbeing which were linked to consumption did not increase by 52% as those who were less than 21 years of age (legal age in the model), and those who were dependent were deemed not to have increased well-being in the model.

Given the lack of knowledge of the price elasticity of cannabis (on frequency, quantity, by age or current use status) and the fact these variables were used to fuel the remainder of the model (i.e. treatment demand, MVA, impact on education, and well-being) the impact of alternate prices were not estimated for the whole model. Nonetheless, if one takes the final values of wellbeing under the legalisation- regulation model, and assumes a 50% price decrease (increase) and a price elasticity of 0.5, the total value of wellbeing is $806 million ($484 million). This would result in a NSB of $404.6 million ($81.8 million). This speculation does not factor in the other impacts of the change in demand for cannabis.

Sensitivity analyses demonstrated that when the actual wellbeing from cannabis is decreased to 50% of the original estimate or removed completely neither the status quo nor the legalised–regulated options have an NSB that is greater than zero. Within the ranges estimated for this CBA, variation of the cost components was unlikely to appreciably impact the final results. It must also be acknowledged that due to lack of data the estimation of wellbeing and the impact on education, and thus income for those who start cannabis use as adolescents, were not estimated with precision. Detailed knowledge of the demand curve for cannabis and long term follow-up of a cohort of individuals are prerequisites to better information.

Data needed to be drawn from a variety of sources such as self-report survey data; peer-reviewed and grey literatures; police, court and treatment data; government documents such as annual reports and budget papers; policy documents on tobacco and alcohol; and finally, key informant interviews. Notwithstanding the multiple sources, methods and assumptions, every attempt was made to be consistent in assumptions and, where possible to use data from Australia. When considering the results and their implications, it is necessary to keep in mind that the context (existing rates of use, price, legal structure, health care system) are relevant and that any major change in any of the characteristics or the assumptions may lead to a different cost structure, and different harms or benefits. For example, the existing patterns of enforcing laws against cannabis use, of providing treatment for cannabis use disorder and rates of cannabis use are all specific to this work. These activities do not take place separately from the socioeconomic conditions, disposable income, the availability of health care and existing preferences and attitudes. The results as they are presented are a function of all of these assumptions, the legal and geographical context and most importantly the legalised–regulated alternative constructed for this analysis.

The uncertainty levels (5 and 95 percentiles) around the mean generated from the Monte Carlo analysis were the same for both models. However, the data used to generate these final results had within it considerably different levels of uncertainty for the two models. For example, there was considerable uncertainty in estimating treatment costs for the status quo option in, for example, the rate of uptake of treatment and duration of treatment and this is also factored into the estimates for the legalised-regulated policy option. There was however, considerably more uncertainty within the legalised-regulated policy option. This additional level amount of uncertainty arises in the uncertainty in the uptake of cannabis use in the legalised model and travels through the model in the high and low estimates for treatment.

The legalised–regulated model was constructed under the premise that cannabis is not a harmless substance but rather a psychoactive drug with the potential for causing dependence in some users, impacting decision-making and ability to drive while under the influence and with the potential for negative health and social effects. As such the framework developed aimed to minimise personal and social harms related to cannabis [14], [38], [89]. In taking such public health perspective attempts were made to incorporate the lessons on the negative effects of advertising, competition, oversupply and low prices from the alcohol and tobacco industries. This meant that there were substantial costs which arose for the legalised–regulated option ($18.96 m for enforcing the legalised–regulated option; $12.6 m for consumer information, prevention and quit programs; and $31.2 m for personal licensing). Other legalised–regulated models may not be so highly regulated, and thus so costly. A different legalised–regulated model would require a new cost benefit analysis.

Anti-competition laws in some countries may prevent the introduction of the proposed monopoly arrangement but there are existing examples, such as the government monopoly of off-licence alcohol sales [16], [41]. It would take strong leadership to ensure such a model was permitted with the primary motivation of promoting public health. The activities by the tobacco and alcohol industries provide many examples of why the responsibility of retailing cannabis should be retained by government or a non-profit organisation [90], [91], [92], [93].

## Conclusion

The results from this CBA of two cannabis policies, one of which is a legalised–regulated option which addresses several public health concerns, starts to redress some of the evidence gaps that arise when making public policy in this area. It will be tempting to turn to the potential revenues and to use them as an argument for legalisation. But it must be reiterated that there is considerable uncertainty around these potential revenues, largely because of the uncertainty as to what would actually happen to the black market [16] and the ability to maintain the price at the current street price. Kilmer et al.(2010) make the point that the price would likely have to fall to undermine the existing black market [16]. This price drop would then negatively impact on government revenues and may lead to increased harmful consumption.

While this study compared two alternative cannabis polices we hope that this paper will prompt other researchers to undertake CBAs across a variety of comparison policy regimes as there is considerable scope for further research in this area. For example, a CBA comparing different countries with different regulatory systems, different patterns of cannabis use and different legalised-regulated regimes and so on would go a long way to addressing many policy questions.

It is noteworthy that the results suggest that either policy will produce a positive net social benefit, and there is little difference between them. This implies that the drivers for policy change are more likely to be politics and public opinion, rather than economic arguments.

## References

[1] UNODC (2010) World Drug Report 2010. United Nations Publication Sales No. E.10.XI.13. 194 p. Available: http://www.unodc.org/documents/wdr/WDR_2010/World_Drug_Report_2010_lo-res.pdf. Accessed July 2011.

[2] European Legal Database on Drugs (2007). European Monitoring Centre for Drugs and Drug Addiction. Available: http://eldd.emcdda.europa.eu/html.cfm/index5769EN.html.

[3] Hughes C, Stevens A (2007) The Effects of Decriminalization of Drug Use in Portugal. The Beckley Foundation Drug Policy Programme. Available: http://www.idpc.net/php-bin/documents/BFDPP_BP_14_EffectsOfDecriminalisation_EN.pdf.pdf.

[4] McLaren J, Mattick R (2006) Cannabis in Australia. Use, supply, harms, and responses. Canbera Drug Strategy Branch, Australian Government Department of Health and Ageing.

[5] Room R, Fischer B, Hall W, Lenton S, Reuter P (2008) Cannabis Policy: Moving beyond stalemate. Oxford: Beckely Foundation.

[6] Englesman E (2003) Cannabis Control: the model of the WHO tobacco control treaty. The International Journal of Drug Policy 14: 217–219.

[7] Wodak A, Reinarman C, Cohen P (2002) Cannabis control: costs outweigh the benefits: For. British Medical Journal 324: 105–106. 11786459 10.1136/bmj.324.7329.105 PMC1121996

[8] Limb M (2012) Ban on recreational drugs is impeding science, says former government drugs adviser. BMJ 344; doi:10.1136/:bmj.e3936. 10.1136/bmj.e3936 22661743

[9] Miron J (2005) The budgetary implications of Marijuana prohibition. Cambridge, MA: Harvard University. Available: http://www.prohibitioncosts.org/mironreport.html.

[10] Wilson J (1991) Drugs and Crime In: Tonry M, Wilson J, editors. Drugs and Crime: Crime and Justice Volume 13: The University of Chicago Books. 574 p.

[11] Wilson J (1990) Against the Legalization of Drugs, Commentary. 89: 21–28.

[12] Hall W, Pacula RL (2003) Cannabis Use and Dependence. Public Health and Public Policy. Cambridge: Cambridge University Press.

[13] MacCoun R, Reuter P (2001) Drug War Herisies Learning from Other Vices, Times and Places. Cambridge, UK: Cambridge University Press.

[14] Rolles S (2009) After the war on drugs: Blueprint for regulations Bristol: Transform Drug Policy Foundation. Transform Drug Policy website. Available: http://www.tdpf.org.uk/downloads/blueprint/Blueprint_exec_summary.pdf. Accessed 2010 January.

[15] Hall W, Lynsky M (2009) The challenges in developing a rational cannabis policy. Current Opinion Psychiatry 22: 258–262. 10.1097/YCO.0b013e3283298f36 19293714

[16] Kilmer B, Caulkins J, Liccardo Pacula R, MacCoun R, Reuter P (2010) Altered State? Assessing how marijuan legalization in California could influence marijuana consumption and public markets. Santa Monica: RAND Drug Policy Research Centre. Available: http://www.rand.org/pubs/occasional_papers/OP315.html. Accessed.

[17] May T, Duffy M, Warburton H, Hough M (2007) Policing cannabis as a Class C drug, An arresting change? York: ICPR, School of Law, King’s College.

[18] May T, Warburton H, Turnbull P, Hough M (2002) Times they are a changing. York: Joseph Rountree Foundation.

[19] Donnelly N, Hall W, Christie P (1999) Effects of the cannabis expiation notice scheme on levels and patterns of cannabis use in South Australia: evidence from the National Drug Strategy Household Surveys 1985–1995. Canberra: DoHA. No.37.

[20] Williams J (2004) The effects of price and policy on marijuana use: what can be learned from the Australian experience? Health Economics 13: 123–137. 14737751 10.1002/hec.796

[21] McCaffrey D, Pacula RL, Han B, Ellickson P (2010) Marijuana use and high school dropout: the influence of unobservables. Health Economics 19: 1281–1299. 19937639 10.1002/hec.1561 PMC2910149

[22] van Ours J, Willams J (2009) Why parents worry: Initiation into cannabis use by youth and their educational attainment. Journal of Health Economics 28: 132–142. 18926578 10.1016/j.jhealeco.2008.09.001

[23] Horwood L, Fergusson D, Hayatbakhshm M, Najman J, Coffey C, et al (2010) Cannabis use and educational achievement: findings from three Australasian cohort studies. Drug and Alcohol Dependence 110: 247–253. 20456872 10.1016/j.drugalcdep.2010.03.008

[24] Mann RE, Adlaf E, Zhao J, Stoduto G, Ialomiteanu A, et al (2007) Cannabis use and self-reported collisions in a representative sample of adult drivers. Journal of Safety Research 38: 669–674. 18054598 10.1016/j.jsr.2007.09.004

[25] Laumon B, Gadegbeku B, Martin J, Biecheler M (2005) Cannabis intoxication and fatal road crashes in France: population based case-control study. British Journal of Medicine 331: 1371–1374. 10.1136/bmj.38648.617986.1F PMC1309644 16321993

[26] Vining A, Weimer D (2010) An assessment of important issues concerning the application of benefit-cost analysis to social policy. Journal of Benefit-Cost Analyisis 1: 1–37.

[27] Sindelar J, Jofre-Bonet M, French M, McLellan A (2004) Cost-effectiveness analysis of addiction treatment: paradoxes of multiple outcomes. Drug and Alcohol Dependence 73: 41–50. 14687958 10.1016/j.drugalcdep.2003.09.002

[28] Drummond M, Sculpher M, Torrance G, O’Brien B, Stoddart G (2005) Methods for the Economic Evaluation of Health Care Programmes, Third Edition. Oxford: Oxford Medical Publications.

[29] Tsuchiya A, Williams A (2001) Welfare economics and economic evaluation. In: Drummond M, McGuire A, editors. Economic Evaluation in Health Care Merging Theory With Practice Oxford: Oxford University Press.

[30] Mishan E, Quah E (2007) Cost benefit analysis, 5th Edition. Abingdon: Routledge.

[31] Pudney S (2010) Drugs policy: what should we do about cannabis? Economic Policy 25: 165–211.

[32] McIntosh E (2010) Introduction. In: McIntosh E, Clarke P, Frew E, Lourviere J, editors. Applied Methods of Cost-Benefit Analysis in Health Care. Oxford: Oxford University Press.

[33] Ableson P (2000) Public Economics Principles and Practice. Sydney: Applied Economics.

[34] NSW Police Force (2007) Cannabis Cautioning Scheme. NSW Government Website. Available: http://www.police.nsw.gov.au/community_issues/drugs/ related_information/cannabis_cautioning_scheme. Accessed 2009 October.

[35] NSW Police Force (2009) Thin Blue Line Information Section Young Offenders Act. Sydney: NSW Police Website. Available:http://www.policensw.com/info/gen/y2.html. Accessed 2009 October.

[36] Caulkins J, Kilmer B, MacCoun R, Pacula R, Reuter P (2011) Design considerations for legalizing cannabis: lessons inspired by analysis of California’s Proposition 19. Addiction 107: 865–871. 21985069 10.1111/j.1360-0443.2011.03561.x

[37] Nadelmann E (1989) Drug Prohibition in the United States: Costs, consequences, and alternatives. Science 245: 939–947. 2772647 10.1126/science.2772647

[38] Haden M (2004) Regulation of illegal drugs: an exploration of public health tools. International Journal of Drug Policy 15: 225–230.

[39] McDonald D, Moore R, Norberry J, Wardlaw G, Ballenden N, et al. (1994) Legislative options for cannabis in Australia. Canberra Commonwealth Department of Human Series and Health.

[40] King County Bar Association (2005) Effective drug control: Toward a new legal framework. Seatle: King County Bar Association. King County Bar Association website. Available: https://www.kcba.org/druglaw/pdf/EffectiveDrugControl.pdf. Accessed 2014 April 4.

[41] Babor T, Caetano R, Casswell S, Edwards G, Giesbrecht N, et al. (2003) Alcohol: No Ordinary Commodity. Oxford: Oxford University Press.

[42] Department of Justice (2009) Becoming a poppy grower. Hobart: Tasmanian Government. Available: http://www.justice.tas.gov.au/poppy/becoming_a_grower. Accessed 2009 August.

[43] Godfrey C (2006) Evidenced based illicit drug policy: the potential contribution of economic evaluation techniques. De Economist 154: 563–580.

[44] Trumbull W (1990) Who has standing in cost-benefit analysis. Journal of Policy Analysis and Management 9: 201–218.

[45] Whittington D, MacRae D (1986) The issue of standing in cost-benefit analysis. The Journal of Policy Analysis and Management 5: 665–682.

[46] Shanahan M (2011) Assessing the economic consequences of two cannabis policy options Sydney: New South Wales. 365 p.

[47] Australian Government Productivity Commission (2007) Report on Government Services 2006 Court Administration. Canberra Available: http://www.pc.gov.au/_data/assets/pdf_file/0006/61674/chapter06.pdf. Accessed 2014 March.

[48] Bureau of Crime Statistics and Research (2006) Court Statistics. Sydney: NSW Government Law Link. BOSCAR website, Available: http://www.bocsar.nsw.gov.au/lawlink/bocsar/ll_bocsar.nsf/pages/bocsar_court_stats.

[49] Passey M, Patete S, Bird G, Bold S, Brooks L, et al (2003) Evaluation of the Lismore MERIT Pilot Program Final Report. Lismore: Northern Rivers University Department of Rural Health. Available: http://www.ncahs.nsw.gov.au/tmp/final_report_MERIT_evaluation.pdf. Accessed 2008.

[50] Martire K, Larney S (2009) Principal drug of concern: An analysis of MERIT and RAD client characteristics and outcomes Sydney: Attorney General’s Department, NSW Government. Available: http://www.merit.lawlink.nsw.gov.au/agdbasev7wr/_assets/merit/m771020l1/issue_7_a4_merit_drug.pdf. Accessed 2010 October.

[51] Australian Bureau of Statistics (2006) Employee Earnings and Hours, Australia, May 2006 Canberra Australian Bureau of Statistics. ABS website. Available: http://www.abs.gov.au/ausstats/abs@.nsf/Previousproducts/6306.0Main%20Features2May%202006?opendocument&tabname=Summar-&tabname=Summary&prodno=6306.0&issue=May%202006&num=&-view=. Accessed 2009.

[52] Shanahan M, Ritter A (2014) Intangible outcomes from a policy change: using contingent valuation to quantify potential stigma from a cannabis offence. Journal of Experimental Criminology. 10: 59–77.

[53] Ngui R, Shanahan M (2010) Cannabis use disorder treatment and associated heath care costs: NSW 2007. Sydney: NDARC.UNSW Autralia. NDARC website. Available: http://www.dpmp.unsw.edu.au/DPMPWeb.nsf/resources/Monograph+16.pdf/$file/DPMP+MONO+20.pdf.

[54] Burns L, Mattick R, Cooke M (2006) The use of record linkage to examine illicit drug use in pregnancy. Addiction 101: 873–882. 16696631 10.1111/j.1360-0443.2006.01444.x

[55] English D, Hulse G, Milne E, Holman C, Bower C (1997) Maternal cannabis use and birth weight: a meta-analysis. Addiction 92: 1553–1560. 9519497

[56] Fergusson D, Horwood L, Northstone K (2002) Maternal use of cannabis and pregnancy outcome. BJOG 109: 21–27. 11843371 10.1111/j.1471-0528.2002.01020.x

[57] Crancer A, Crancer A (2010) The Involvement of Marijuana in California Fatal Motor Vehicle Crashes 1998–2008. Available: http://druggeddriving.org/pdfs/CAMJStudyJune2010.pdf. Accessed 2014 April 4.

[58] Drummer O, Gerostamoulos J, Batziris H, Chu M, Caplehorn J, et al (2004) The involvement of drugs in drivers of motor vehicles killed in Australian road traffic crashes. Accident Analysis and Prevention 36: 239–248. 14642878 10.1016/s0001-4575(02)00153-7

[59] Fergusson D, Horwood L (2001) Cannabis use and traffic accidents in a birth cohort of young adults. Accident Analysis & Prevention 33: 703–711. 11579972 10.1016/s0001-4575(00)00082-8

[60] Moore TH, Zammit S, Lingford-Hughes A, Barnes TR, Jones PB, et al (2007) Cannabis use and risk of psychotic or affective mental health outcomes: a systematic review. Lancet 370: 319–328. 17662880 10.1016/S0140-6736(07)61162-3

[61] Australian Institute of Health and Welfare (2008) 2007 National Drug Strategy Household Survey. Detailed Findings Canberra: Australian Institute of Health and Welfare.2010 National Drug Strategy Household Survey report (AIHW)^∧^.

[62] Carr V, Neil A, Halpin S, Holmes S, Lewin T (2003) Costs of schizophrenia and other psychoses in urban Australia: findings from the low prevalence (psychotic) disorders study. Australian New Zealand Journal of Psychiatry 37: 31–40. 12534654 10.1046/j.1440-1614.2003.01092.x

[63] Tolkien II Team (2006) Tolkien II. A needs-based, costed stepped care model for Mental Health Services. Darlinghurst, NSW, Australia: World Health Organisation Collaborating Centre for Classification in Mental Health.

[64] Pudney S, Badillo C, Bryan M, Burton J, Conti G, et al. (2006) Estimating the size of the UK illicit drug market. In: Singleton N, Murray R, Tinsley T, editors. Measuring different aspects of problem drug use: methodological developments: Home Office Online Report. Available: http://www.homeoffice.gov.uk/publications/science-research-statistics/research-statistics/crime-research/hoor1606. Accessed 2008 January.

[65] Rhodes W, Langenbahn S, Kling R, Scheiman P (1997) What America’s users spend on illegal drugs, 1988–1995. Rockville MD: Office of National Drug Control Policy.

[66] Hickman M, Vickerman P, Macleod J, Lewis G, Zammit S, et al. (2010) If cannabis caused schizophrenia–how many cannabis users may need to be prevented in order to prevent one case of schizophrenia? England and Wales calculations. Addiction 104 1856–1861. 10.1111/j.1360-0443.2009.02736.x 19832786

[67] Teesson M, Slade T, Swift W, Mills K, Memedovic S, et al (2012) Prevalence, correlates and comorbidity of DSM-IV Cannabis Use Disorders in Australia: Findings of the 2007 National Survey of Mental Health and Wellbeing Australian & New Zealand Journal of Psychiatry. 46: 1182–1192. 10.1177/0004867412460591 22984111

[68] Mann RE, Stoduto G, MacDonald S, Brands B, editors (2008) Cannabis use and driving: implications for public health and transport policy. Lisbon: European Monitoring Centre for Drugs and Addiction (EMCDDA).

[69] Pacula RL (2010) Examining the impact of Marijuana legalisation on harms associated with marijuana use. Santa Monica: Rand. RAND wsite. http://www.rand.org/pubs/working_papers/WR769.html. Accessed 2014 April.

[70] Access Economics (2008) The health of nations: the value of a statistical life. Canberra: Australian Safety and Compensation Council. Safework Australia website. Available: http://www.safeworkaustralia.gov.au/AboutSafeWorkAustralia/WhatWeDo/Publications/Documents/330/TheHealthOfNations_Value_StatisticalLife_2008_PDF.pdf. Accessed 2014 April 4.

[71] Leigh A, Ryan C (2008) Estimating returns to education using different natural experiment techniques. Economics of Education Review 27: 149–160.

[72] Zarkin G, Cates S, Bala M (2000) Estimating the willingness to pay for drug abuse treatment: A pilot study Journal of Substance Abuse Treatment. 18: 149–159. 10.1016/s0740-5472(99)00030-6 10716098

[73] French M, Salome H, Sindelar J, McLellan A (2002) Benefit-cost analysis of addiction treatment: methodological guidelines and empirical application using the DATCAP and ASI. Health Services Research 37: 433–455. 12036002 10.1111/1475-6773.031 PMC1430361

[74] Weimer D, Vining A, Thomas R (2009) Cost-benefit analysis involving addictive goods: Contingent valuation to estimate willingness-to pay for smoking cessation. Health Economics 18: 181–202. 18566968 10.1002/hec.1365

[75] Boys A, Marsden J, Strang J (2001) Understanding reasons for drug use amongst young people: a functional perspective. Health Education Research 16: 457–469. 11525392 10.1093/her/16.4.457

[76] Phillips B, Burns L (2008) NSW Drug Trends 2008 Findings from the Illicit Drug Reporting System (IDRS) Sydney: NDARC, UNSW Australia. NDARC website Available: http://ndarc.med.unsw.edu.au/project/illicit-drug-reporting-system-idrs. Accessed 2009 February.

[77] Roads and Traffic Authority (2010) Licensing. Sydney: NSW Government. NSW Government website. Available: http://www.rta.nsw.gov.au/licensing/. Accessed 2009 October.

[78] California Department of Public Health (2010) Medical Marijuana Program Fees. State of California. Available: http://www.cdph.ca.gov/programs/MMP/Pages/MMPFees.aspx. Accessed 2011 July.

[79] Communities Office of Liquor Gaming and Racing (2010) Underage drinking offences. Sydney: New South Wales Government. Available: http://www.olgr.nsw.gov.au/youth_fines.asp. Accessed 2009 October.

[80] Ministerial Council on Drug Strategy (2005) Australian National Tobacco Strategy 2004–2009: Guide to Planning and Investing in Tobacco Control. Canberra Available: http://www.health.gov.au/internet/main/publishing.nsf/Content/E955EA2B5D178432CA256FD30017A522/$File/tobacco_planning.pdf. Accessed 2010 December 1.

[81] Australian Institute of Health and Welfare (2008) 2007 National Drug Strategy Household Survey. First Results Canberra: Australian Institute of Health and Welfare. Available: www.aihw.gov.au/publication-detail/?id=6442468084. Accessed 2008 November.

[82] NSW Industrial Relations (2008) NSW public sector awards and agreements 2008. Sydney NSW Governement. NSW Industrial Relations (2008) NSW public sector awards and agreements 2008. Accessed 2008 July.

[83] Australian Competition and Consumer Commission (2008) Report of the ACCC inquiry into the competitiveness of retail prices for standard groceries. Canberra: Australian Competition and Consumer Commission. http://transition.accc.gov.au/content/index.phtml?itemId=838251. Accessed 2010 January.

[84] Geis G (2005) Chop-chop: The illegal cigarette market in Australia. Canberra: Centre for Tax System Integrity. Available: http://regnet.anu.edu.au/sites/default/files/ROP2.pdf. Accessed 2008 March.

[85] Cameron L, Williams J (2001) Cannabis, alcohol and cigarettes: substitutes or complements? The Economic Record 77: 19–34.

[86] Williams J, Pacula RL, Chaloupka FJ, Wechsler H (2004) Alcohol and marijuana use among college students: economic compliments or substitutes. Health Economics 133: 825–843. 10.1002/hec.859 15362176

[87] MacCoun R (2010) Estimating the non-price effects of legalization on cannabis consumption. Santa Monica: RAND. RAND website. Available: http://www.rand.org/pubs/working_papers/WR767.html. Accessed 2014 April 4.

[88] Weatherburn D, Jones C, Donnelly N (2003) Prohibition and Cannabis Use in Australia: A Survey of 18- to 29-year-olds. The Australian and New Zealand Journal of Criminology 36: 77–93.

[89] Wood E, Werb D, Fischer B, Hart C, Wodak A, et al. (2010) Tools for debate: US Federal Government data on cannabis prohibition. Vancouver: International Centre for Science in Drug Policy. International Centre for Science in Drug Policy website. Available: http://www.icsdp.org/research/publications/toolsfordebate.aspx. Accessed 2011 January.

[90] Callard C, Thompson D, Collishaw N (2005) Transforming the tobacco market: why the supply of cigarettes should be transferred from for-profit corporations to non-profit enterprises with a public health mandate Tobacco Control. 14: 278–283. 10.1136/tc.2005.011353 PMC1748051 16046692

[91] World Health Organization (2005) WHO Framework Convention on Tobacco Control. Geneva, Switzerland: WHO Document Production Services. Available: http://www.who.int/tobacco/framework/WHO_FCTC_english.pdf. Accessed 2010 September.

[92] Freeman B, Chapman S, Rimmer M (2008) The case for plain packaging of tobacco products. Addiction 103: 580–590. 18339104 10.1111/j.1360-0443.2008.02145.x

[93] Cancer Council Victoria (2010) Tobacco in Australia: facts and issues a comprehensive online resource. Melbourne. Tobacco in Australia website Available: http://www.tobaccoinaustralia.org.au/chapter-11-advertising/11-3-federal-legislation. 2010 Accessed November.

